# Comparison of Long-Term Oncologic Outcomes Between Surgical T4 and T3 in Patients Diagnosed With Pathologic Stage IIA Right Colon Cancer

**DOI:** 10.3389/fonc.2022.931414

**Published:** 2022-07-14

**Authors:** Youngbae Jeon, Kug Hyun Nam, Seok Won Choi, Tae Sik Hwang, Jeong-Heum Baek

**Affiliations:** ^1^ Division of Colon and Rectal Surgery, Department of Surgery, Gil Medical Center, Gachon University College of Medicine, Incheon, South Korea; ^2^ Songdo General Surgery Clinic, Incheon, South Korea; ^3^ Pyeongtaek St. Mary’s Hospital, Pyeongtaek, South Korea; ^4^ Yonsei Hang General Surgery Clinic, Incheon, South Korea

**Keywords:** surgical stage, colon cancer, oncologic outcomes, survival, recurrence

## Abstract

**Purpose:**

T stage plays an important role in the classification of subgroups in stage II colon cancer. Patients with pathologic T4 are at high risk of recurrence and it is recommended to include adjuvant chemotherapy in the treatment plan, while this is not necessary in pathologic T3. There is a discrepancy between the surgical T stage (sT), as determined by the surgeon in the operative field, and pathologic T stage (pT). The pathologic stage is considered a standard prognostic factor, but it has not been established whether the surgical stage has an oncologic impact. The aim of this study was to compare oncologic outcomes between sT4 and sT3 in pathologic stage IIA right colon cancer.

**Methods:**

Between January 2005 and December 2018, there were 354 patients who underwent right hemicolectomy performed by a single surgeon (JHB) at a tertiary hospital. The data from these patients were retrospectively collected and analyzed. Only those patients with pathologic stage IIA (pT3N0M0) right colon adenocarcinomas were included in this study. Patients with mucinous carcinoma, signet ring cell carcinoma, squamous cell carcinoma, or hereditary colon cancer, and who had emergent surgery were excluded. Finally, 86 patients were included in this study. The patients were categorized, according to their surgical records, into either the sT4 group (n=28) or the sT3 group (n=58).

**Results:**

There were no statistical differences between the two groups in terms of age, sex, body mass index, comorbidities, cancer location, histologic grade, lymphovascular invasion, perineural invasion, number of harvested lymph nodes, and adjuvant chemotherapy. The 5-year overall survival rate was significantly different between the sT4 and sT3 groups (92.6% vs. 97.7%, p=0.024). In addition, the 5-year disease-free survival rate was significantly different between the sT4 and sT3 groups (88.6% vs. 97.7%, p=0.017). In the multivariate Cox regression analysis, a classification of sT4 was a significant independent predictive factor for recurrence (*p* = 0.023).

**Conclusions:**

Long-term oncologic outcomes have shown significant differences between surgical T4 and T3 in pathologic stage IIA right colon cancer patients. Further large-scale, multicenter studies are required to verify the clinical impact of the surgical staging.

## Introduction

Colorectal cancer is the third most commonly diagnosed malignant disease and the second leading cause of cancer-related death worldwide (1). The current standard practice for non-metastatic colon cancer is to perform either only surgery (including endoscopic resection) or surgery followed by adjuvant chemotherapy, depending on the pathologic staging results. The most widely used staging system for malignant tumors, including colon cancer, is the Tumor Nodes Metastasis (TNM) staging classification from the American Joint Committee on Cancer (AJCC) (2). It is widely accepted that TNM staging is a significant prognostic factor in oncologic outcomes (3, 4). The importance of tumor staging systems has increased over the past several decades as tailored therapies, which rely on an accurate understanding of the disease progression, become more prominent (5). The T stage plays an important role in the classification of cancer subgroups, especially in patients with stage II (T3 or T4, N0) colon cancer. Patients with pathologic T4 stage II colon cancer are recommended for adjuvant chemotherapy due to the high risk of recurrence, while patients with pathologic T3 stage II colon cancer are not (6, 7). Therefore, it is essential to distinguish patients in stage T4 from those in stage T3 to ensure they receive adjuvant therapy to improve their chances of survival.

In clinical practice, the surgical T stage (sT), which is determined by the surgeon in the operative field, is typically recorded. However, there is a discrepancy in staging between the sT and pathologic T stage (pT). The sT and pT staging were reported to be equivalent in 78.48% of colon cancer cases (8). Intraoperatively tumors suspected to be sT4 are often finally diagnosed as pT3 tumors. Thus, there are concerns regarding the underestimation of pT4. The pT stage has been considered a standard prognostic factor, but it is not established whether the sT stage has an oncologic impact. The aim of this study was to compare oncologic outcomes between sT4 and sT3 in pathologic stage IIA (pT3N0M0) of right colon cancer to explore the oncologic impact of the sT stage.

## Materials and Methods

### Patient Selection and Surgical T Stage

Between January 2005 and December 2018, 354 patients underwent right hemicolectomy for right colon cancer, performed by a single surgeon (JHB) at a tertiary referral hospital, Gil Medical Center, Incheon, South Korea. All relevant data were retrospectively collected and analyzed. Only the medical records of patients with pathologic stage IIA (pT3N0M0) right colon adenocarcinomas were included in this study. Patients with mucinous carcinoma, signet ring cell carcinoma, squamous cell carcinoma, hereditary colon cancer, recurrent colon cancer, history of other malignancies, those who underwent emergent surgery, and those who had no information of surgical T stage in medical records were excluded. Finally, 86 patients were included in this study. The operative records of all patients were reviewed to obtain the sT, with 28 patients categorized into the sT4 group and 58 into the sT3 group. The pT was reported according to the AJCC TNM staging system, 7^th^ and 8^th^ editions (2, 9). The sT was assessed by a senior colorectal surgeon during surgery and checked against the gross specimen after resection. The sT3 was assessed by following surgical finding; The surgeon can detect the location of primary tumor under direct vision with the lesion appearing on the serosa of colorectal wall, but there were no invasion through the serosa, and no macroscopic adherence between tumor and either peritoneum or organ in surgical field. The sT4 was assessed by following surgical finding; The surgeon can detect the serosal invasion of the primary tumor, or the tumor macroscopically adheres or invades to adjacent organ or structure in surgical field (**Table 1**). Patient selection was performed by searching the Clinical Research Data Warehouse system at our institution and detailed data were retrospectively collected from the electronic medical records at the Gil Medical Center. Institutional review board approval was obtained from the Ethics Committee of the same hospital (approval no. GCIRB2021-349). Additionally, to identify the equivalency between sT3, 4 and pT3, 4, the distribution was investigated at all pTNM stages of right colon cancer.

**Table 1:** Comparison between the pathologic T stage* and surgical T stage** in primary tumor (T) staging of patients with right colorectal cancer.

Primary tumor (T)	Pathologic T stage^*^	Surgical T stage^**^
Tx	Primary tumor cannot be assessed	Primary tumor cannot be assessed
T0	No evidence of primary tumor	No evidence of primary tumor
Tis	Carcinoma in situ, intramucosal carcinoma	Practically difficult to distinguish among Tis, T1, and T2
T1	tumor invades the submucosa	
T2	Tumor invades the muscularis propria	
T3	Tumor invades through the muscularis propria into pericolorectal tissues	The surgeon can detect the location of primary tumor under direct vision with the lesion appearing on the serosa of colorectal wall, but there were no invasion through the serosa, and no macroscopic adherence between tumor and either peritoneum or organ in surgical field.
T4	Tumor invades through the visceral peritoneum (T4a) or invades or adheres to adjacent organ or structure (T4b)	The surgeon can detect the serosal invasion of the primary tumor, or the tumor macroscopically adheres or invades to adjacent organ or structure in surgical field.

^*^Pathological T stage is described based on the Tumor Nodes Metastasis staging classification from The American Joint Committee on Cancer, 7^th^ and 8^th^ editions.

^**^Surgical T stage is determined by the colorectal surgeon depending on the intraoperative findings.

### Assessment Parameters

Selected clinical features, including age, sex, body mass index (BMI), American Society of Anesthesiology (ASA) score, comorbidities, tumor location, preoperative tumor marker levels, adjuvant chemotherapy, and histopathological outcomes were compared between the sT4 and sT3 groups. Overall survival was calculated from the date of surgery for colon cancer to the date of any cause of death, or loss of follow up. Disease-free survival was defined as the duration of time from the date of surgery for colon cancer to the date of local recurrence, distant metastasis, death, or loss of follow up.

### Patient Follow-Up

The Gil Medical Center has preexisting follow up protocols in place for patients with cancer, therefore all patients diagnosed with stage IIA colon cancer were followed up every 3 months with serial carcinoembryonic antigen (CEA) levels and physical examination. Abdominopelvic computed tomography scans were performed annually for the first 5 years after surgery. Surveillance colonoscopies were performed every 2 - 3 years for the first 5 years. Patients with stage IIA colon cancer who have high risk factors for recurrence, including poorly differentiated histology, lymphovascular invasion, perineural invasion, perforation, obstruction, positive resection margin, or less than 12 harvested lymph nodes, are usually treated with adjuvant FOLFOX (folinic acid, fluorouracil, and oxaliplatin) chemotherapy for 12 cycles according to the current guidelines (10, 11).

### Statistical Analysis

Continuous variables were analyzed using the Mann–Whitney U test, and categorical variables were compared using Pearson’s chi-square test and Fischer’s exact test. Survival analysis was performed using the Kaplan–Meier method and the log-rank test. Cox’s proportional hazard model was used for multivariate analysis to identify factors predicting the prognosis. Significant differences between the two groups for each variable were defined as a two-tailed *p*-value < 0.05. All statistical analyses were performed using IBM SPSS Statistics for Windows, version 23 (IBM Corp., Armonk, N.Y., USA)

## Results

### Patient Demographics

The baseline patient demographics are presented in **Table 2**. The median age was 69 years (range: 40 - 87 years) in the sT4 group and 66 years (range: 23 - 92 years) in the sT3 group. Fourteen (50.0%) patients in the sT4 group and 33 (56.9%) in the sT3 group, respectively, were men. The most common tumor location in both the groups was the ascending colon (60/86, 69.8%). Preoperative bowel obstruction and perforation were present in 11 (13.1%) and 8 (9.5%) patients, respectively. Twelve (42.9%) patients received adjuvant chemotherapy in the sT4 group and 24 (41.4%) patients were administered adjuvant chemotherapy in the sT3 group. There were no statistically significant differences between the two groups in terms of baseline clinical features.

**Table 2:** Baseline patient demographics.

Variables	sT4 (n=28) n (%)	sT3 (n=58) n (%)	*p*
Age, years (range)	69 (40-87)	66 (23-92)	0.764
Sex			0.646
Male	14 (50.0)	33 (56.9)	
Female	14 (50.0)	25 (43.1)	
BMI, kg/m^2^ (range)	21.8 (17.3-28.0)	22.9 (16.4-33.6)	0.253
ASA score			0.683
1	2 (7.1)	3 (5.2)	
2	23 (82.1)	45 (77.6)	
3	3 (10.7)	10 (17.2)	
Comorbidities			
Cardiovascular disease	13 (16.4)	33 (56.9)	0.489
Pulmonary disease	2 (7.1)	6 (10.3)	0.483
Diabetes mellitus	8 (28.6)	15 (25.9)	0.800
Location of tumor			0.945
Cecum	4 (14.3)	11 (19.0)	
Ascending colon	20 (71.4)	40 (69.0)	
Hepatic flexure	2 (7.1)	4 (6.9)	
Proximal transverse colon	2 (7.1)	3 (5.2)	
Preoperative CEA			1.000
≥5 ng/mL	4 (14.3)	9 (15.5)	
<5 ng/mL	23 (82.1)	45 (77.6)	
Unknown	1 (3.6)	4 (6.9)	
Presenting findings			
Bowel obstruction	5 (17.9)	6 (10.3)	0.327
Bowel perforation	3 (10.7)	5 (8.6)	0.712
Adjuvant chemotherapy	12 (42.9)	24 (41.4)	1.000

sT, surgical T stage; BMI, body mass index; ASA, American Society of Anesthesiologists; CEA, carcinoembryonic antigen.

### Histopathological Characteristics

The histopathological characteristics are presented in **Table 3**. Moderately differentiated was the most common histological grade found in this study (71/86, 82.6%). Lymphovascular invasion and perineural invasion were found in 8 (28.6%) and 3 (10.7%) patients in the sT4 group, respectively, and 15 (25.9%) and 5 (8.6%) patients in the sT3 group, respectively. The median number of harvested lymph nodes was 26 (range: 8 - 60) in the sT4 group and 25 (range: 5 - 74) in the sT3 group. There were 3 (10.7%) patients in the sT4 group and 4 (6.9%) patients in the sT3 group who had high microsatellite instability. No statistically significant differences were identified between the two groups in terms of histopathological characteristics (**Table 3**).

**Table 3:** Histopathological characteristics.

Variables	sT4 (n=28) n (%)	sT3 (n=58) n (%)	*p*
Histologic grade			1.000
Well differentiated	2 (7.1)	6 (10.3)	
Moderately differentiated	24 (85.7)	47 (81.0)	
Poorly differentiated	2 (7.1)	5 (8.6)	
Unknown	0 (0)	1 (1.7)	
Lymphovascular invasion			0.800
Present	8 (28.6)	15 (25.9)	
Absent	20 (71.4)	43 (74.1)	
Perineural invasion			0.679
Present	3 (10.7)	5 (8.6)	
Absent	19 (67.9)	35 (60.3)	
Unknown	6 (21.4)	18 (31.0)	
Harvested lymph node (range)	26 (8-60)	25 (5-74)	0.761
Microsatellite status			0.881
High	3 (10.7)	4 (6.9)	
Low or stable	12 (42.9)	26 (44.8)	
Unknown	13 (16.4)	28 (48.3)	

### Survival Outcomes and Predictive Factors

**Figure 1:**
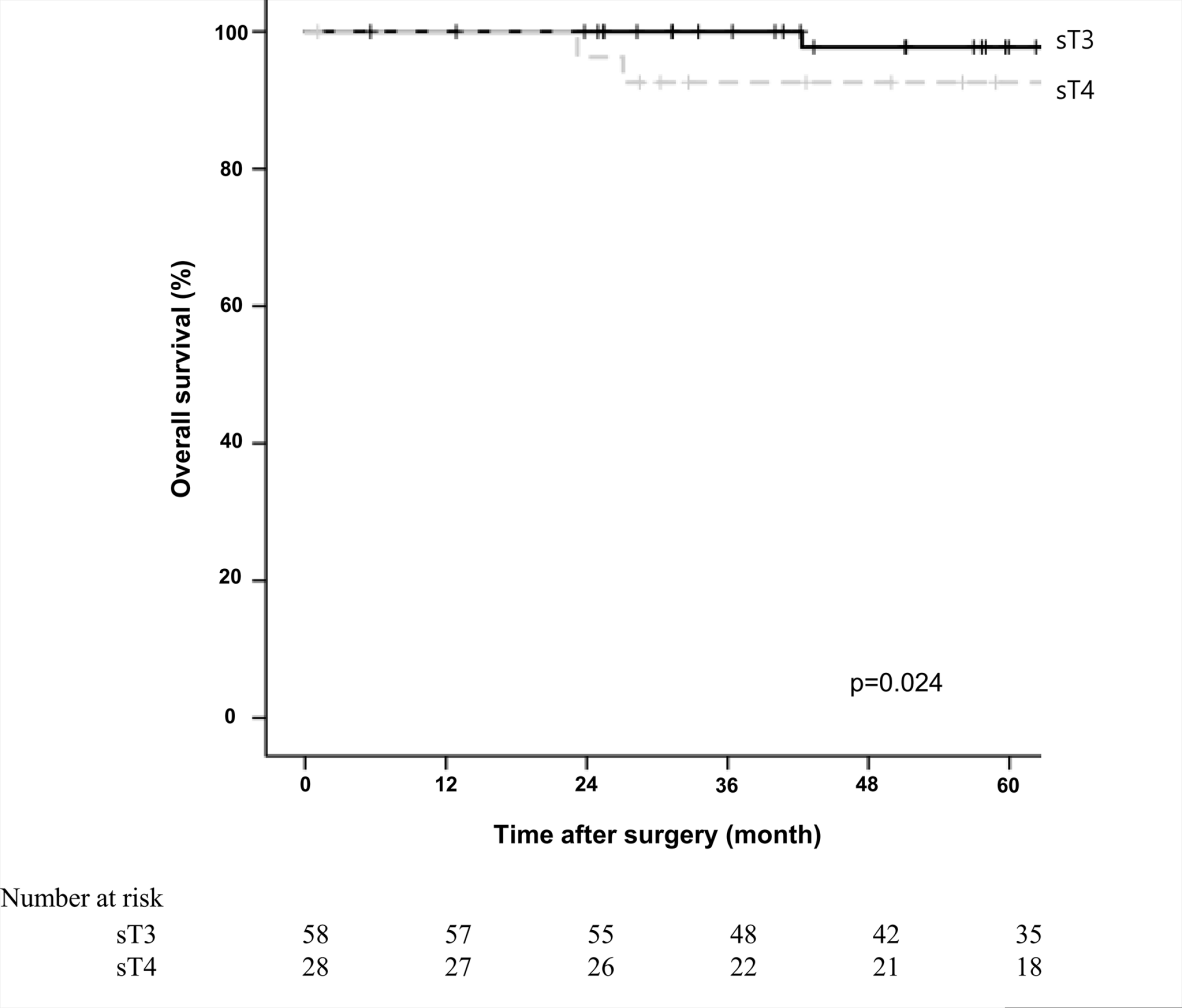
Comparing overall survival for right colon adenocarcinoma between surgical stage T4 (sT4) and surgical stage T3 (sT3) in patients with pT3N0M0 by Kaplan-Meier curve. The 5-year overall survival rate of sT4 group was significantly lower than that of sT3 groups according to log-rank test (92.6% vs. 97.7%, *p*=0.024).

**Figure 2:**
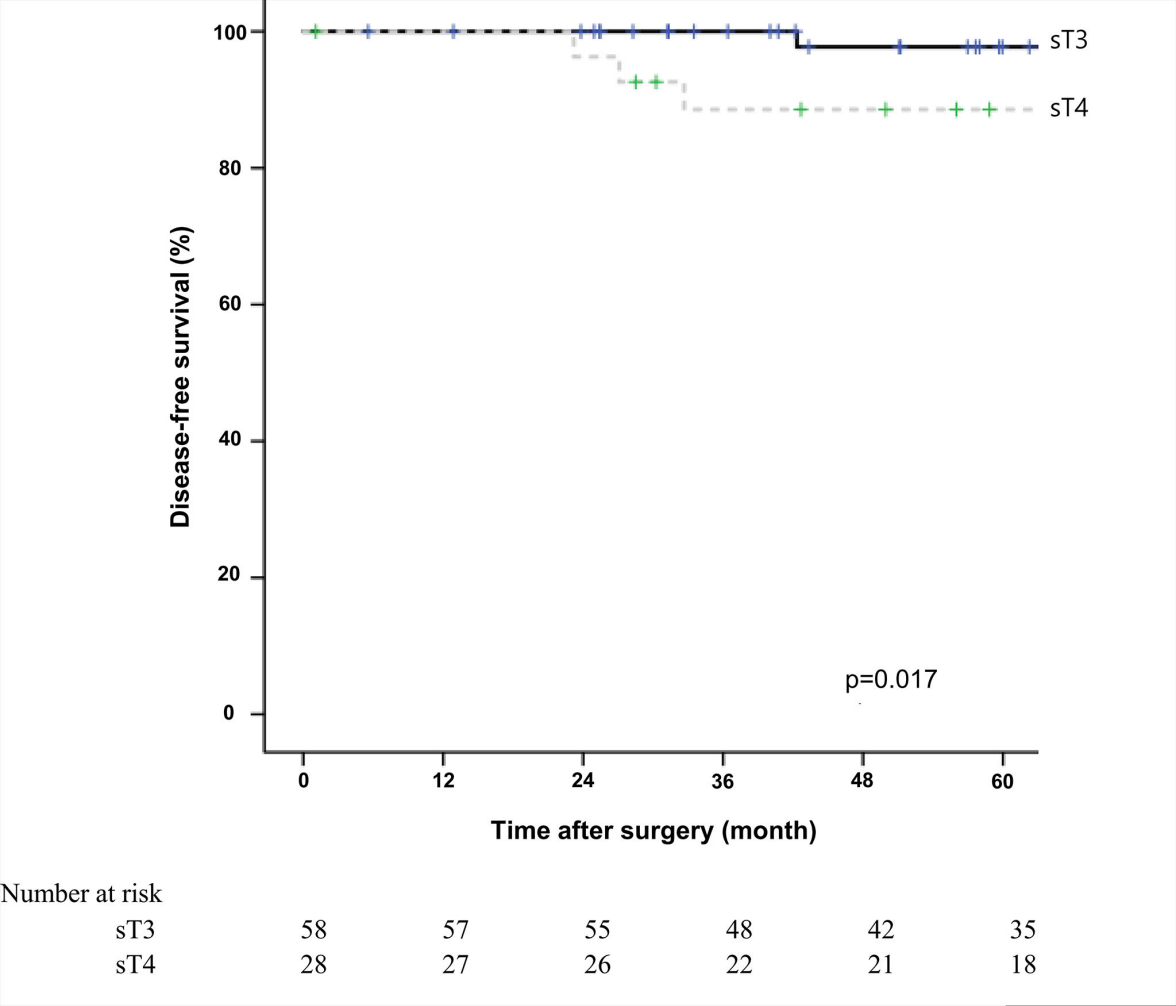
Comparing disease-free survival for right colon adenocarcinoma between surgical stage T4 (sT4) and surgical stage T3 (sT3) in patients with pT3N0M0 by Kaplan-Meier curve. The 5-year disease-free survival rate of sT4 group was significantly lower than that of sT3 groups according to log-rank test (88.6% vs. 97.7%, *p*=0.017).

The 5-year overall survival rate was significantly different between the sT4 and sT3 groups (92.6% vs. 97.7%, *p*=0.024; **Figure 1**). The 5-year disease-free survival rate also showed a significant difference between the two groups (88.6% vs. 97.7%, *p*=0.017; **Figure 2**). The overall recurrence rate was 8.1% (7/86). The median follow-up period was 92.5 months in the sT4 group and 79.4 months in the sT3 group. Multivariate analysis of the overall survival by the Cox proportional hazard model showed no statistically significant predictive factors, but sT4 presented a trend toward significance [hazard ratio (HR), 8.007; 95% confidence interval (CI): 0.806 - 74.023, *p* = 0.067]. For disease-free survival, only sT4 showed a statistically significant difference (HR, 7.303; 95% CI: 1.314 - 40.596, *p* = 0.023) (**Table 4**).

**Table 4:** Univariate log-rank test and multivariate Cox proportional hazards model for survival.

Variables	Univariate log-rank test	Multivariate Cox regression analysis^*^		
	*p*	HR	95% CI	*p*
Overall survival				
Age ≥ 65 years	0.143	4.308	0.389-47.680	0.234
Male sex	0.972			
BMI ≥ 25	0.659			
Perforation	0.380			
Obstruction	0.400			
sT4	0.024^**^	8.007	0.866-74.023	0.067
Poorly differentiated	0.481			
Lymphovascular invasion	0.561			
Harvested LN < 12	0.010^**^	5.054	0.492-51-860	0.173
Adjuvant chemotherapy	0.553			
Disease-free survival				
Age ≥ 65 years	0.157	0.354	0.045-2.760	0.322
Male sex	0.484			
BMI ≥ 25	0.222			
Perforation	0.512			
Obstruction	0.548			
sT4	0.017^**^	7.303	1.314-40.596	0.023^**^
Poorly differentiated	0.781			
Lymphovascular invasion	0.424			
Harvested LN < 12	0.025^**^	2.938	0.304-28.376	0.352
Adjuvant chemotherapy	0.123	0.199	0.031-1.287	0.090

^*^After all variables that showed p ≥ 0.2 in univariate analysis were removed, multivariate Cox regression analysis was performed.

^**^Statistical significance, p < 0.05.

HR, hazard ratio; CI, confidence interval; BMI, body mass index; sT4, surgical stage T4; LN, lymph nodes.

### Equivalency of the Pathologic T Stages and the Surgical T Stages

Distribution of the pathologic T3, 4 stages and the surgical T3, 4 stages in all pathologic TNM stages of right colon cancer was presented in **Table 5**. The patients who were recorded as sT3 were finally diagnosed with 87.8% of pT3, and 12.2% of pT4, respectively. The patients who were recorded as sT4 were finally diagnosed with 70.4% of pT3, and 29.6% of pT4.

**Table 5:** Distribution of the pathologic T3, 4 stages and the surgical T3, 4 stages in all pathologic TNM stages of right colon cancer.

	pT3, n (%)	pT4, n (%)	Total, n (%)
sT3, n (%)	65 (87.8)	9 (12.2)	74 (100)
sT4, n (%)	76 (70.4)	32 (29.6)	108 (100)

## Discussion

The importance of tailored therapies in cancer management has increased in the last decades and as such, many studies have focused on identifying predictive clinicopathological factors. From this perspective, our study’s aim of identifying the oncologic impact of the surgical T stage is in line with the current trends in cancer research. In our study, the sT4 group showed significantly lower overall survival rates and worse disease-free survival rates than those in the sT3 group. Furthermore, in the multivariate analysis, a staging of sT4 was the only significant predictive factor for recurrence. This implies that some of the patients who are diagnosed with pT3 may actually be pT4 and have been incorrectly classified. As mentioned above, surgical and pathologic T stages are matched in only 78.48% of colorectal cancer cases, and approximately 20% of patients are at risk of under or over treatment (8). Compton et al. insisted that peritoneal invasion is often underdiagnosed by pathologists when they only assessed by routine histologic examination, because documentation of peritoneal invasion required extensive sampling and serial sectioning (12). In the studies with cytologic examination of serosal scrapings, malignant cells presented 26% of tumor specimens which diagnosed as pT3 by histologic examination alone (13, 14). In our study, while the proportion of matching values of sT3 and pT3 was 87.8%, that of sT4 and pT4 was only 29.6%. It is assumed that the serosal lesions, which were only inflammatory changes, were also overestimated, because the surgeon recorded it as sT4 if there were any suspicions of serosal involvement or macroscopic tumor adherence.

It is also possible that sT4 itself, that is, the macroscopic tumor adherence, is an independent prognostic factor, even though the nature of tumor adherence represents a histologically malignant extension or only an inflammatory change. Several studies have emphasized the oncologic impact of macroscopic tumor adherence as a predictive factor for colorectal cancer (15–17). A study from Australia evaluated the associations between colorectal tumor adherence and other clinicopathological features in 268 patients with tumor adherence among 2504 patients who underwent colorectal surgeries (15). Adherent tumors were independently associated with pelvic recurrence (HR: 1.8, 95% CI: 1.2 - 2.7, *p* = 0.007), systemic recurrence (HR: 1.7, 95% CI: 1.1 - 2.4, *p* = 0.009), and poor overall survival (HR: 1.6, 95% CI: 1.3 - 2.0, *p* < 0.001) in rectal cancer, although there was no association between tumor adherence and survival in colon cancer. Another study proposed a revised pT category (r-pT) in colorectal cancer patients with a discrepancy between the surgical T stage and pathologic T stage (16). The patients with pT3 and sT4 were reclassified into r-pT4a and the patients with pT4a and sT4b were reclassified into r-pT4b. The r-pT stage showed superior predictive outcomes compared to the standard pT stage (Harrell’s C: 0.668 vs. 0.636, *p* = 0.002) and presented an independent prognostic factor in multivariable regression analysis (HR: 1.846, 95% CI: 1.566 - 2.176, *p* < 0.001). This study was proposed as an alternative to overcome the weakness of the pathologically-oriented staging system. The last brief study also found that macroscopic serosal invasion, defined as tumor extent or colloid changes protruding from the surface of the serosa, was an independent risk factor for recurrence in multivariable survival analysis (HR: 4.750, 95% CI: 1.381 - 16.334, *p* = 0.013) among 375 patients with stage IIA colon cancer (17). They insisted that macroscopic serosal invasion in the operative findings may complement the shortcomings of the standard pathologic T category if there were inconsistencies between surgical and pathologic T stages. This is similar to our results and the assumptions of our study.

The surgical T stage is in accordance with the AJCC staging system but has a drawback in that it is difficult to distinguish between sTis, sT1, and sT2 in operative findings, while it is possible to distinguish between sT3 and sT4. Other studies have suggested the following criteria for the surgical T stage: sT1 lesions were diagnosed when the lesion appeared normal, and this assessment was combined with the preoperative auxiliary examination; sT2 lesions were diagnosed when the lesion was mobile on the muscle layer of the colorectal wall; sT3 lesions were diagnosed when the tumor did not invade through the serosa, and the lesion appeared nodular on the serosal layer of the colorectal wall; sT4a lesions were diagnosed when serosal involvement was visible; and sT4b lesions were diagnosed when tumor directly invaded or was adherent to other organs or structures (16). In our study, we only focused on the surgical T stage in patients with pathological stage IIA. We did not find sTis, sT1, or sT2 lesions in pT3 patients; that is, all patients in pT3 were staged as sT3 or sT4. Furthermore, we believed that pathological assessment was sufficient for the stratification of T stages below T3, while operative findings were more important in distinguishing between T3 and T4 because T4 requires information regarding the relationship between the tumor and adjacent organs or structures.

The present study had several limitations. First, it was a retrospective, single-center analysis with a small population, which can lead to selection bias. The results of the multivariate Cox regression analysis were somewhat unrefined due to a small sample size (n=86), and a small number of events. Because there were quite a few patients who had no information of surgical T stage in their medical records, only small number of patients included in this study. It is the main drawback of our research. Second, we did not divide sT4a and sT4b, although the classification has been adapted from the 7^th^ edition of the AJCC TNM staging classification. There were a couple of records filled with sT4a or sT4b, however, most of the records were filled with only just sT4. This is also considered a limitation of retrospective nature in this study. Third, we confined our study to patients with right colon adenocarcinoma to exclude the oncologic influence of tumor sidedness. Other tumor locations, including sigmoid colon cancer and rectal cancer, should also be evaluated. Fourth, the proportion of matching values between sT stage and pT stage was too low. Especially, only 30% of patients with sT4 were finally diagnosed with pT4, whereas 88% of patients with sT3 were finally diagnosed with pT3. Even considering the tendency of the surgeon to overestimate the surgical stages, it is necessary to assess the surgical T stage with clearer criteria. Finally, we did not check external validation. It is crucial to prove a prediction model’s reproducibility and generalizability. Therefore, further researches should be prospective designed, and multicenter with big data studies. Nonetheless, this study has a strength in terms of identifying novel prognostic factors in the era of individual cancer therapy. In particular, the surgical T stage may estimate the patients’ prognosis as a complementary factor to the pathologic findings. Furthermore, proper adjuvant chemotherapy or close follow up may be considered in patients with sT4 right colon cancer who are diagnosed with pathologic stage IIA through multidisciplinary discussion with pathologic review.

## Conclusions

Comparative analysis of long-term oncologic outcomes showed significant differences between the sT4 and sT3 groups in pathologic stage IIA of patients with right colon adenocarcinoma. Surgical T stage may assist in distinguishing patients with a high risk of recurrence who are required adjuvant treatment to complement the surgical treatment. Our results suggest that multidisciplinary discussions, including those between surgeons and pathologists, are essential to manage colon cancer patients, especially when there is a discrepancy between the surgical and pathologic T stage. However, the limitations with small sample size and retrospective nature analysis should be overcome. Further large-scale prospective multicenter studies are needed to verify the oncologic impact of sT.

## Data Availability Statement

The raw data supporting the conclusions of this article will be made available by the authors, without undue reservation.

## Ethics Statement

The studies involving human participants were reviewed and approved by Institutional review board approval was obtained from the Ethics Committee of Gil Medical Center, Gachon University College of Medicine (approval no. GCIRB2021-349). Written informed consent for participation was not required for this study in accordance with the national legislation and the institutional requirements.

## Author Contributions

YJ – data analysis and interpretation, drafting of manuscript and critical revision. KN – acquisition of data, data analysis and interpretation, and manuscript drafting. SC – acquisition of data. TH – acquisition of data. JH-B – conception and design of study, acquisition of data, critical revision of manuscript. All authors have approved the final version of the manuscript.

## Conflict of Interest

The authors declare that the research was conducted in the absence of any commercial or financial relationships that could be construed as a potential conflict of interest.

## Publisher’s Note

All claims expressed in this article are solely those of the authors and do not necessarily represent those of their affiliated organizations, or those of the publisher, the editors and the reviewers. Any product that may be evaluated in this article, or claim that may be made by its manufacturer, is not guaranteed or endorsed by the publisher.

## Abbreviations

AJCC, American Joint Committee on Cancer; ASA, American Society of Anesthesiologist; BMI, body mass index; CEA, carcinoembryonic antigen; pT, pathological T stage; sT, surgical T stage; TNM, Tumor Nodes Metastasis.
